# Can central-venous oxygen saturation be estimated from tissue oxygen saturation during a venous occlusion test?

**DOI:** 10.5935/0103-507X.20220023-en

**Published:** 2022

**Authors:** Claudio da Silva Zachia Alan, Alexandre Augusto Pinto Lima, Jan Bakker, Gilberto Friedman

**Affiliations:** 1 Postgraduate Program in Pulmonology Sciences, Universidade Federal do Rio Grande do Sul - Porto Alegre (RS), Brazil.; 2 Department of Intensive Care, Erasmus MC, University Medical Center - Rotterdam, The Netherlands.

**Keywords:** Spectroscopy, near-infrared, Oxygen saturation, Oxygen consumption, Critically illness

## Abstract

**Objective:**

To test whether tissue oxygen saturation (StO_2_) after a venous occlusion test estimates central venous oxygen saturation (ScvO_2_).

**Methods:**

Observational study in intensive care unit patients. Tissue oxygen saturation was monitored (InSpectra Tissue Spectrometer Model 650, Hutchinson Technology Inc., MN, USA) with a multiprobe (15/25mm) in the thenar position. A venous occlusion test in volunteers was applied in the upper arm to test the tolerability and pattern of StO_2_ changes during the venous occlusion test. A sphygmomanometer cuff was inflated to a pressure 30mmHg above diastolic pressure until StO_2_ reached a plateau and deflated to 0mmHg. Tissue oxygen saturation parameters were divided into resting StO_2_ (r-StO_2_) and minimal StO_2_ (m-StO_2_) at the end of the venous occlusion test. In patients, the cuff was inflated to a pressure 30mmHg above diastolic pressure for 5 min (volunteers’ time derived) or until a StO_2_ plateau was reached. Tissue oxygen saturation parameters were divided into r-StO_2_, m-StO_2_, and the mean time that StO_2_ reached ScvO_2_. The StO_2_ value at the mean time was compared to ScvO_2_.

**Results:**

All 9 volunteers tolerated the venous occlusion test. The time for tolerability or the StO_2_ plateau was 7 ± 1 minutes. We studied 22 patients. The mean time for StO_2_ equalized ScvO_2_ was 100 sec and 95 sec (15/25mm probes). The StO_2_ value at 100 sec ([100-StO_2_] 15mm: 74 ± 7%; 25mm: 74 ± 6%) was then compared with ScvO_2_ (75 ± 6%). The StO_2_ value at 100 sec correlated with ScvO_2_ (15 mm: R^2^ = 0.63, 25mm: R^2^ = 0.67, p < 0.01) without discrepancy (Bland Altman).

**Conclusion:**

Central venous oxygen saturation can be estimated from StO_2_ during a venous occlusion test.

## INTRODUCTION

Failure of oxygen delivery leads to an increase in the extraction ratio of oxygen from the blood, resulting in a decrease in mixed venous saturation (SmvO_2_). Measurement of SmvO_2_ is an established form of monitoring global DO_2_/VO_2_ balance (oxygen extraction) in adult intensive care.^(1)^

However, the measurement of SmvO_2_ requires access to blood from the pulmonary artery, which is an invasive hemodynamic monitoring technique. Monitoring central venous oxygen saturation (ScvO_2_) has been advocated as a simple method to evaluate changes in the relationship between supply and demand oxygen in various clinical settings.^(2,3)^ Therefore, ScvO_2_ measurements have become an established form of monitoring systemic tissue oxygenation in critically ill patients.^(4,5)^ Its measurement requires a central venous line that should reside in the superior vena cava. Thus, blood sampling collected in this location reflects systemic oxygenation mainly from the upper part of the body. Venous oxygen saturation differs among several organ systems since they have different metabolic rates.^(6,7)^

However, measurements of ScvO_2_ are not without hazards and can be infeasible in certain scenarios. Therefore, it would be useful to find a noninvasive technique to estimate ScvO_2_. Near-infrared spectroscopy (NIRS) offers a technique for continuous, noninvasive, bedside monitoring of tissue oxygenation.^(8)^ Near-infrared spectroscopy uses the principles of light transmission and absorption to measure the concentrations of hemoglobin noninvasively in tissues and provides a global assessment of oxygenation in all vascular compartments (arterial, venous and capillary). It has been used to assess forearm skeletal muscle oxygenation during induced reactive hyperemia in healthy adults, and it produced reproducible measurements of tissue oxygenation during both arterial and venous occlusive events.^(9-11)^ Using the venous occlusion test (VOT) method, NIRS can be applied to measure changes in tissue oxygen saturation (StO_2_) by following the changes in the concentrations of oxygenated and deoxygenated hemoglobin (HbO_2_ and Hb). In this method, a pneumatic cuff is inflated to a pressure above the diastolic and below the systolic pressure for a few seconds. Such pressure blocks venous outflow but does not prevent arterial inflow, unlike the standard vascular occlusion test. As a result, venous blood volume and pressure increase,^(11,12)^ and the increased pooling of venous blood causes an increase in Hb.

Thus, it is reasonable to assume that NIRS will reflect this change by decreasing the StO_2_ value. In view of these observations, the aim of this study was to test the hypothesis that NIRS with VOT could be used to estimate ScvO_2_ in a population of critically ill patients.

## METHODS

### Study population

This prospective observational study was conducted in the intensive care unit (ICU) of a university hospital with 33 beds of the Erasmus Medical Center - Rotterdam, Netherlands. We enrolled consecutive adult (>18 years) critically ill patients within 24 hours of ICU admission who had undergone initial resuscitation and stabilization. All patients were mechanically ventilated and had a central catheter with the tip placed in the superior vena cava. The ICU has single-person closed rooms, and the ambient temperature in each patient’s room was individually and actively set at 22°C. None of the patients had elevated bilirubin levels. We also recruited healthy volunteers with no history of receiving any vasoactive medication. The volunteers were instructed not to consume caffeine-containing drinks until after the experiments. The institutional review board approved the study. Each patient (or his or her relative) and healthy volunteer provided written informed consent.

### Measurements

#### StO_2_-derived tissue oxygenation

StO_2_-derived tissue oxygenation was continuously monitored using an InSpectra Tissue Spectrometer Model 650 (Hutchinson Technology Inc., Hutchinson, MN, USA) with a multiprobe (15 and 25mm) over the thenar eminence.

#### Venous occlusion in healthy volunteers

Venous occlusion (VO) in healthy volunteers was designed to investigate how long a person can tolerate venous pooling of VO and to study the pattern of StO_2_ changes during VO. Venous occlusion was performed by arresting forearm blood flow using a conventional sphygmomanometer pneumatic cuff. All volunteers were seated with their arm rested on a table at the heart level, and StO_2_-derived tissue oxygenation was continuously monitored with the probe over the thenar eminence. The cuff was placed around the upper arm and was inflated to a pressure approximately 30mmHg greater than diastolic pressure until StO_2_ reached a plateau line or compression was not tolerated. The plateau or tolerance was considered the completion of the occlusion period, and then the cuff was rapidly deflated.

VO-derived StO_2_ parameters were divided into two components: resting StO_2_ (r-StO_2_) and minimal StO_2_ (m-StO_2_) at the end of venous occlusion.

#### Venous occlusion in patients

A blood sample (1mL of blood after withdrawal of dead-space blood) was collected from the central line placed in the superior vena cava to measure ScvO_2_ before initiation of the VOT. All measurements were made using a cooximeter. All patients had to have arterial saturation greater than 92% (Maximo Pulse Oximetry). Following the recording of ScvO_2_, StO_2_-derived tissue oxygenation was continuously monitored with the probe over the thenar eminence, and the VO test was then performed by arresting forearm blood flow using a conventional sphygmomanometer pneumatic cuff. The cuff was placed around the upper arm and was inflated to a pressure approximately 30mmHg greater than diastolic pressure for 5 minutes (time limit derived from the healthy volunteers) or until a plateau line on the screen was reached. On the completion of the occlusion period, the occluding cuff was rapidly deflated. VO-derived StO_2_ parameters were divided into three components: resting StO_2_ values (r-StO_2_), the minimum StO_2_ value at the end of venous occlusion (m-StO_2_), and the time that StO_2_ reached the same ScvO_2_ of the patient (StO_2_ = ScvO_2_). The StO_2_ value at the mean time, when StO_2_ equals ScvO_2_, was subsequently compared with ScvO_2_.

The first measurement was performed within 24 hours of intensive care admission after hemodynamic stability was obtained (MAP > 65mmHg, and no change in vasopressor use for 2 hours) every 24 hours thereafter until Day 3.

#### Statistics

A sample size of 19 patients was estimated for a correlation coefficient of 0.6 (two tailed alpha = 0.05, beta = 0.20). The results are presented as the mean ± standard deviation, unless otherwise specified. A paired t test was conducted to estimate significant differences after a normality test (Kolmogorov-Smirnov test). Bivariate correlation was used to determine whether r-StO_2_, m-StO_2_, 100-StO_2_ and ScvO_2_ were linearly related to each other. To compare StO_2_ and ScvO_2_, we calculated bias, systemic disagreement between measurements (mean difference between two measurements) and precision (the random error in measuring [standard deviation of bias]).

The 95% limits of agreement were arbitrarily set, in accordance with Bland and Altman, as the bias ± 1.96 standard deviations.^(13)^ A p value < 0.05 was considered statistically significant (SPSS version 15.0, Chicago, IL).

## RESULTS

### Healthy volunteers

**Figure 1:**
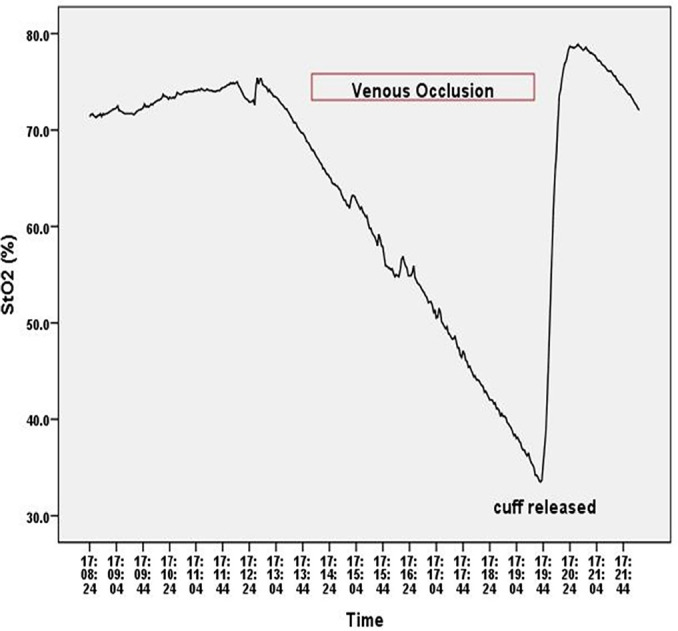
An example of the tissue oxygen saturation pattern during a venous occlusion test in a healthy volunteer. StO_2_ - tissue oxygen saturation.

All volunteers (n = 9) tolerated the occlusion test well, and the average tolerance time was 7 ± 1 minutes. The VO using a pneumatic cuff resulted in an immediate decrease in StO_2_ in all volunteers in a linear pattern, and the release of the occlusion was followed by a rapid increase in StO_2_ (Figure 1). Considering that all healthy volunteers tolerated well more than 5 minutes of VO, we used this time length (5 minutes) as a convenient VOT time to be applied in patients prevented from communicating tolerance.

### Critically ill patients

We included 22 ICU patients (Table 1). All patients were under analgesia or sedation, and we performed 42 measurements on 3 consecutive days. Table 2 shows the average ScvO_2_ and VO-derived StO_2_ (15mm and 25mm) for all patients. In four patients, equalization of StO_2_ and ScvO_2_ was never obtained.

**Table 1:** Patient characteristics

Variables	
Age (year)	57 ± 18
Male/Female	8/14
APACHE II	20 ± 7
SOFA	7 ± 4
Main admission category	
Sepsis/severe sepsis	6
Septic shock	6
Shock, no sepsis	5
No shock, no sepsis	5
Physiological parameters (42 measurements)	
Blood lactate (mmol/L)	1.6 ± 1.2
Capillary refill (sec)	9.1 ± 8.1
Central temperature (C°)	36.7 ± 1.1
Mean arterial pressure (mmHg)	82 ± 17
Oximetry arterial saturation (%)	96 ± 3

APACHE - Acute Physiology and Chronic Health Evaluation; SOFA - Sequential Organ Failure Assessment. Results expressed as mean ± standard deviation or n.

**Figure 2:**
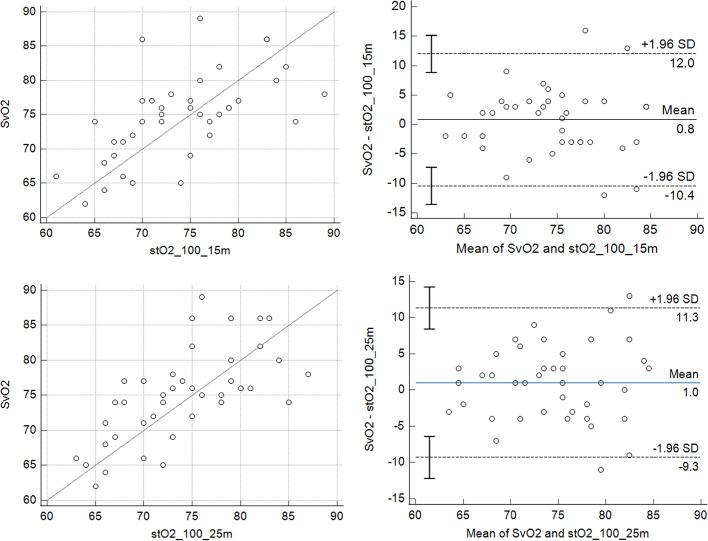
The correlation and Bland Altman analysis between central venous oxygen saturation and tissue oxygen saturation (probes 15mm and 25mm) after 100 seconds of the venous occlusion test in patients. SvO_2_ - venous oxygen saturation; StO_2_ - tissue oxygen saturation; SD - standard deviation.

**Figure 3:**
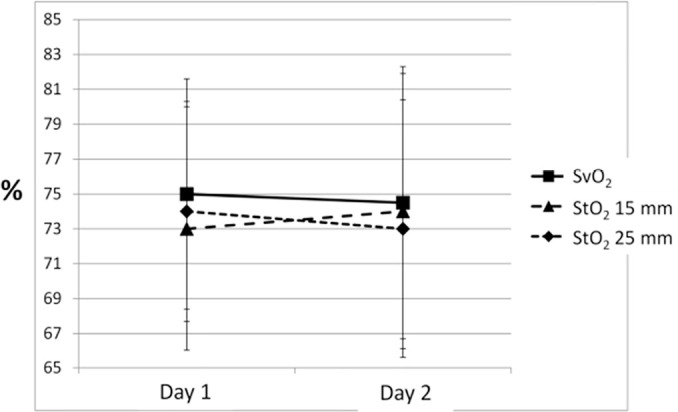
Tissue oxygen saturation after 100 sec. of venous occlusion for both probes tracks central venous oxygen saturation on day two. SvO_2_ - venous oxygen saturation; StO_2_ - tissue oxygen saturation.

The mean time duration required for both variables to reach the same value was 100 seconds and 95 seconds for StO_2_ 15mm and 25mm, respectively. Using 100 seconds as a time reference, we used the StO_2_ descend line of VO to select the exact StO_2_ value after 100 seconds of VO (100-StO_2_). That value was then registered and compared with ScvO_2_. Table 2 shows ScvO_2_ and VO-derived StO_2_ parameters stratified by the StO_2_ probe type. 100-StO_2_ was significantly correlated with ScvO_2_, and Bland Altman analysis revealed relevant agreement for both probes (Figure 2). There were no differences between ScvO_2_ and 100-StO_2_ values on Day 1 and Day 2 (Day 3 was not analyzed, as only two patients had a third measurement - Figure 3).

We found a significant correlation between ScvO_2_ and r-StO_2_ (15mm probe: R^2^ = 0.46, p = 0.003; 25mm probe: R^2^ = 0.36, p = 0.02) or m-StO_2_ (15mm probe: R^2^ = 0.41, p = 0.008; 25mm probe: R^2^ = 0.32, p = 0.04). At rest, ScvO_2_ values were lower than StO_2_ values but higher than minimal StO_2_ values (Table 2).

## DISCUSSION

The main finding of our study is that peripheral venous oxygen saturation (SvO_2_) after a VO test moderately correlates with ScvO_2_. In addition, we have shown that the time to equalization of both values was close to 100 sec. This may be a method that can assess peripheral StO_2_ noninvasively and which can be repeated every few minutes and help to estimate ScvO_2_ or its trend.^(11,12,14,15)^. In particular, Yoxall et al. and Wardle et al. two decades ago investigated peripheral venous hemoglobin saturation measured by using near infrared spectroscopy after venous occlusion of the forearm with partial venous occlusion to track changes in the balance between global oxygen demand and consumption in preterm infants and adults.^(11,12,15)^

The first part of the study evaluated VOT tolerability in healthy volunteers. We observed that the VO test until a StO_2_ plateau line was obtained was well tolerated even after more than 7 minutes. Based on that result, we used identified a convenient VO time length (5 minutes) to be used in the patients. We assumed that this time length was reasonable to obtain the equalization of StO_2_ and ScvO_2_ in our patients. Similar to Yoxall et al., we have shown that the VOT was safe and feasible.^(11,12)^

We found a moderate correlation between StO_2_ and ScvO_2_ after 100 sec of the VOT. The mean time required for the StO_2_ value to equalize with the ScvO_2_ for most patients value was rather short (~100 sec), much less than the predefined 5 minutes VO duration, which makes the VO test rather accurate and safe. Central venous oxygen saturation and 100-StO_2_ were well correlated without significant discrepancy for both probes, which strengthens the use of StO_2_ after a VOT as an estimation of ScvO_2_.

Both r-StO_2_ and m-StO_2_ were significantly correlated with ScvO_2_, but the relationship was somewhat weak. At rest, other investigators have shown a similar finding, particularly in nonseptic patients in whom the oxygen extraction capabilities are preserved.^(10,16)^ Experimental and clinical studies on hypovolemic shock show that StO_2_ levels correlate with systemic flow variables.^(10,17-20)^ However, there is uncertainty regarding this correlation when the patient is septic due to altered oxygen extraction capabilities, as has been shown previously after stagnant ischemia.^(16)^ In fact, half of our patients were septic, which could explain the limited correlation found in our study.

Prior to the VOT, ScvO_2_ values were lower than r-StO_2_, which is explained by two mechanisms. First, the peripheral arterial compartment is intact before stagnant ischemia.^(21)^ Second, critically ill patients, mainly septic patients, do show high StO_2_ because reduced cellular extraction of oxygen is common.^(22,23)^ In turn, after the VOT, the m-StO_2_ values decreased considerably. We speculate that the StO_2_ falls below the ScvO_2_ during VOT because StO_2_ is related not only to oxygen consumption but also to the reactive vasoconstriction that occurs during vascular occlusion.^(24)^ Therefore, the decrease in StO_2_ is due to two factors: muscle VO_2_ and reactive vasoconstriction, which induces the sharpest decline in StO_2_ (mainly the arterial component).

We did not evaluate whether dynamic changes in ScvO_2_ (ex. after a fluid challenge) were followed by similar changes in 100-StO_2_ or m-StO_2_. However, 100-StO_2_ tracked ScvO_2_ on Day 2. We could not evaluate this relationship on Day 3 because only two patients were followed on the third day. This result, although imperfect to evaluate acute changes in ScvO_2_, suggests that the 100-StO_2_/ScvO_2_ relation is not affected by the clinical condition of the patient. It could be interesting to study this relationship in patients during acute resuscitation, as our patients were partially or fully resuscitated, as one can see by normal ScvO_2_ and lactate mean values.

It is important to emphasize that the results of tissue saturation found in the thenar muscle of one hand may not be reproducible in the other if the conditions of demand and/or oxygen consumption are different.

The regional or peripheral venous saturation value can only be extrapolated as being SvcO_2_ if the patients are to mirror at least the venous saturation of the upper part of the body.

This study has some limitations that should be acknowledged. First, measurements during changes in the oxygen extraction ratio were not made in this study to investigate whether StO_2_ after a VOT would rapidly track the changes in ScvO_2_. It is well known that significant changes in StO_2_ could occur after an intervention that induces ischemia and reperfusion.^(25)^ Our focus, however, was to assess the relation between StO_2_ after a VOT and ScvO_2_ in a single moment. Second, changes in ambient temperature at each patient’s bedside were not measured. However, the ICU consists of one-person closed rooms, and the ambient temperature in each patient room was individually controlled at 22°C. Last, the duration of the VO test (5 minutes) was based on the volunteers’ results, but we do not know whether it is appropriate in heterogeneous disease conditions. There is no support in the literature to show which method of VOT is superior or more reliable to assess the relation between ScvO_2_ and peripheral SvO_2_. In addition, we arbitrarily chose to use the StO_2_ value after 100 seconds of VOT based on our findings in healthy volunteers. Our strategy must be tested in different settings. Therefore, the results of our study cannot be extended to other studies that have addressed the ScvO_2_/StO_2_ relation.(11,12,14,15)

We established the usefulness of the monitoring of StO_2_ after a venous occlusion test in critically ill patients. We found that ScvO_2_ and StO_2_ correlate, but StO_2_ levels are accompanied by alterations in the peripheral circulation, indicating that StO_2_ abnormalities are related to regional hemodynamics and macrohemodynamics. Thus, it is not surprising that the correlation between both parameters is imperfect but still of clinical usefulness.

## CONCLUSION

In conclusion, this study has shown the feasibility of frequent noninvasive measurements of peripheral venous oxygen saturation as an estimation of central venous oxygen saturation after a venous occlusion test. However, it is not yet advisable to recommend predicting absolute values of venous oxygen saturation for any given patient based solely on the noninvasive measurement of tissue oxygen saturation after a venous occlusion test, as the correlation was only moderate. Further clinical studies are required before it is considered a useful adjunct to the clinical monitoring setting.
